# Community-Based Participatory Research Strategies to Collect, Analyze, and Disseminate Qualitative Data

**DOI:** 10.1093/geroni/igaa057.1866

**Published:** 2020-12-16

**Authors:** Roma Hanks, Hosik Min, Shoon Lio, Brandon Moss, Sarah Wraight, Denise Lewis

**Affiliations:** 1 University of South Alabama, Mobile, Alabama, United States; 2 University of Georgia, Athens, Georgia, United States

## Abstract

Project 1: Community Health Advocates participated in a photovoice project that evolved into an invited installation at the Alabama Contemporary Art Center, giving statewide exposure to health issues in communities in South Alabama that had been identified as those with the highest health disparities. The context of the project was a multi-generational approach to community health advocacy. Project 2: This project focused on employing CBPR methods in Cambodian and Laotian immigrant communities in South Alabama to discover barriers to disaster preparedness, response, and recovery and to better understand the intersections of culture, spirituality, and social justice along the path to community empowerment and resiliency. Community engagement in the development of emergency plans is typically at the organization-to-organization level. This paper analyzes multi-generational, multi-disciplinary, multi-method approaches using qualitative data to build effective strategies for advocacy through community engagement in research.

